# Ultra-low-dose chest CT imaging of COVID-19 patients using a deep residual neural network

**DOI:** 10.1007/s00330-020-07225-6

**Published:** 2020-09-03

**Authors:** Isaac Shiri, Azadeh Akhavanallaf, Amirhossein Sanaat, Yazdan Salimi, Dariush Askari, Zahra Mansouri, Sajad P. Shayesteh, Mohammad Hasanian, Kiara Rezaei-Kalantari, Ali Salahshour, Saleh Sandoughdaran, Hamid Abdollahi, Hossein Arabi, Habib Zaidi

**Affiliations:** 1 Division of Nuclear Medicine and Molecular Imaging, Geneva University Hospital, CH-1211 Geneva, Switzerland; 2 Department of Radiology Technology, Shahid Beheshti University of Medical, Tehran, Iran; 3 Department of Biomedical Engineering and Medical Physics, Shahid Beheshti University of Medical Sciences, Tehran, Iran; 4 Department of Physiology, Pharmacology and Medical Physics, Alborz University of Medical Sciences, Karaj, Iran; 5 Department of Radiology, Arak University of Medical Sciences, Arak, Iran; 6 Rajaie Cardiovascular, Medical & Research Center, Iran University of Medical Science, Tehran, Iran; 7 Department of Radiology, Alborz University of Medical Sciences, Karaj, Iran; 8 Department of Radiation Oncology, Shahid Beheshti University of Medical Sciences, Tehran, Iran; 9 Department of Radiologic Sciences and Medical Physics, Faculty of Allied Medicine, Kerman University of Medical sciences, Kerman, Iran; 10 Geneva University Neurocenter, Geneva University, CH-1205 Geneva, Switzerland; 11 Department of Nuclear Medicine and Molecular Imaging, University of Groningen, University Medical Center Groningen, Groningen, Netherlands; 12 Department of Nuclear Medicine, University of Southern Denmark, Odense, Denmark

**Keywords:** COVID-19, Tomography X-ray computed, Deep learning, Artificial intelligence

## Abstract

**Objectives:**

The current study aimed to design an ultra-low-dose CT examination protocol using a deep learning approach suitable for clinical diagnosis of COVID-19 patients.

**Methods:**

In this study, 800, 170, and 171 pairs of ultra-low-dose and full-dose CT images were used as input/output as training, test, and external validation set, respectively, to implement the full-dose prediction technique. A residual convolutional neural network was applied to generate full-dose from ultra-low-dose CT images. The quality of predicted CT images was assessed using root mean square error (RMSE), structural similarity index (SSIM), and peak signal-to-noise ratio (PSNR). Scores ranging from 1 to 5 were assigned reflecting subjective assessment of image quality and related COVID-19 features, including ground glass opacities (GGO), crazy paving (CP), consolidation (CS), nodular infiltrates (NI), bronchovascular thickening (BVT), and pleural effusion (PE).

**Results:**

The radiation dose in terms of CT dose index (CTDI_vol_) was reduced by up to 89%. The RMSE decreased from 0.16 ± 0.05 to 0.09 ± 0.02 and from 0.16 ± 0.06 to 0.08 ± 0.02 for the predicted compared with ultra-low-dose CT images in the test and external validation set, respectively. The overall scoring assigned by radiologists showed an acceptance rate of 4.72 ± 0.57 out of 5 for reference full-dose CT images, while ultra-low-dose CT images rated 2.78 ± 0.9. The predicted CT images using the deep learning algorithm achieved a score of 4.42 ± 0.8.

**Conclusions:**

The results demonstrated that the deep learning algorithm is capable of predicting standard full-dose CT images with acceptable quality for the clinical diagnosis of COVID-19 positive patients with substantial radiation dose reduction.

**Key Points:**

*• Ultra-low-dose CT imaging of COVID-19 patients would result in the loss of critical information about lesion types, which could potentially affect clinical diagnosis.*

*• Deep learning–based prediction of full-dose from ultra-low-dose CT images for the diagnosis of COVID-19 could reduce the radiation dose by up to 89%.*

*• Deep learning algorithms failed to recover the correct lesion structure/density for a number of patients considered outliers, and as such, further research and development is warranted to address these limitations.*

**Electronic supplementary material:**

The online version of this article (10.1007/s00330-020-07225-6) contains supplementary material, which is available to authorized users.

## Introduction

The emergence of a novel coronavirus in December 2019 in Wuhan, China, known as severe acute respiratory syndrome coronavirus 2 (SARS-CoV-2) was recognized as a global public health concern by the World Health Organization (WHO) [1]. SARS-CoV-2 disease 2019 or COVID-19 is an infectious disease that affects the upper and lower respiratory tract and induces mild to severe respiratory syndromes, including pneumonia [2]. Real-time reverse transcription-polymerase chain reaction (RT-PCR) is considered the standard method for COVID-19 diagnosis but is prone to a number of limitations, including the time of preparation and false-positive and false-negative rates in different clinical samples [3]. Conversely, early studies confirmed that computed tomography (CT) is a feasible approach for COVID-19 diagnosis [4]. Until recently, a wide range of clinical studies have been conducted on the feasibility of CT findings in the early detection and management of COVID-19 patients. However, there are still considerable knowledge gaps in the recognition of CT features linked to COVID-19 [4, 5].

As CT examinations account for the major cause of radiation exposure to the general public from diagnostic medical imaging procedures, the development of low-dose CT imaging protocols is highly desirable. A recent study demonstrated that DNA double-strand breaks and chromosome aberrations increased in patients undergoing a standard-dose CT examination while no effect on human DNA was detected in patients undergoing low-dose CT scans [6]. Although a plethora of hardware and software technological advances in CT dose reduction have been reported, including high-sensitivity detectors, new automatic exposure control (AEC) systems, adaptive x-ray tube voltage, and new image reconstruction algorithms, CT is still not a low-dose imaging modality [7]. Therefore, the level of radiation exposure from this modality is still a matter of concern [8]. Task-specific low-dose imaging protocols devised in both academic and corporate settings were adopted in a clinical setting [9]. Zhou et al [10] suggested a low-dose CT protocol enabling to significantly reduce the dose-length product (DLP) and effective dose (ED) without sacrificing signal-to-noise ratio (SNR) and contrast-to-noise ratio (CNR). Nevertheless, converting from conventional full-dose to low-dose CT imaging protocols is not a simple task owing to the fear of increasing the false-positive rate due to the elevated level of noise and missing anatomical structures.

A number of professional societies, scientists, and clinicians proposed appropriate low-dose CT protocols for COVID-19 [11–14]. However, these protocols are not widely deployed in clinical centers for the same abovementioned reasons. Clinicians and radiologists often tend to use established protocols employing full-dose CT imaging and often lack time or are reluctant to develop or adopt new protocols, especially during emergency situations, such as during the COVID-19 outbreak.

In addition to conventional denoising approaches [15, 16], a number of deep learning algorithms have been proposed for medical image analysis [17–19], PET [20], and SPECT [21] denoising as well as CT image denoising and enhancement of image quality [10, 22–25]. Yang et al [22] applied a generative adversarial network (GAN) with the Wasserstein distance and perceptual loss to denoise low-dose CT images. In another study, Kim et al [23] investigated the effect of different loss functions on convolutional neural network (CNN)–based image denoising performance using task-based image quality assessment for various signals and dose levels. Shin et al [24] compared the image quality of low-dose CT images obtained using a deep learning–based denoising algorithm with low-dose CT images reconstructed using filtered backprojection (FBP) and advanced modeled iterative reconstruction (ADMIRE). They reported that deep learning techniques achieved better noise properties compared with FBP and ADMIRE reconstructions of low-dose CT images. In this work, we aimed to use deep learning algorithms on ultra-low-dose COVID-19 CT images to generate high-quality images for a comparable diagnostic accuracy with full-dose CT images.

## Materials and methods

### Data acquisition

This retrospective study was approved by the ethics committees of the participating centers. Written consent was waived with approval. We included 1141 volumetric chest CT exams from 9 medical centers, among which 312 volumetric CT images were from PCR-positive COVID-19 patients*.* COVID-19 patients were collected from three centers and various scanner models, including Emotion 16 (Siemens Healthcare), NeuViz Dual (Neusoft Medical Systems), and Optima CT580 (GE Healthcare). All CT images were acquired in each center using the same protocol and were reconstructed using a filtered backprojection (FBP) algorithm (Table 1).

**Table 1:** Acquisition parameters of full-dose and low-dose chest CT protocols

Parameters	Full-dose CT	Low-dose CT
CTDI_vol_ (mGy)	6.5 (4.16–10.5)	0.72 (0.66–1.03)
Voltage (kVp)	100–120	90
Tube current (mA)	100–150	20–45
Pitch factor	1.3–1.8	0.75

### Ultra-low-dose CT simulation

Based on Beer-Lambert law (*I* = *I*_0_ exp(− ∫ *μ*(*e*, *x*)*dx*)), the incident flux level of the ultra-low-dose scan (*I*_0_) can be calculated by adequately scaling the incident flux level of the corresponding full-dose scan. According to the physics of CT transmission data (Eq. 1), we simulated ultra-low-dose CT projection data from full-dose projections in the sinogram domain by adding a statistically independent Poisson noise distribution and a Gaussian noise distribution.

1 $$ \hat{I}=\mathrm{Poisson}\left({I}_0\right)+\mathrm{Gaussian}\left({m}_e,{\sigma}_e^2\right) $$

where *Î* is the measured noisy signal recorded in the detector channels, and *I*_0_ is the mean number of photons passing through the patient determined based on a linear relationship with tube current (mAs). *m*_*e*_ and ${\sigma}_e^2$ are the mean and variance of the electronic noise, respectively. The whole procedure is as follows:

1. Converting Hounsfield units (HUs) to linear attenuation coefficients according to tube voltage in the full-dose image (${\mu}_{\mathrm{tissue}}=\frac{HU\times \left({\mu}_{\mathrm{water}}-{\mu}_{\mathrm{air}}\right)}{1000}+{\mu}_{\mathrm{water}}$),
2. Generating projection data (*p*_*sd*_) from the attenuation map (*μ*_tissue_) using the Radon transform on the full-dose image with the following setups: parallel beam geometry and 1080 projection angles in one rotation,
3. Converting projection data to the transmission data, i.e., *T*_*sd*_=exp( − *p*_*sd*_),
4. Generating ultra-low-dose transmission data by multiplying ultra-low-dose scan incident flux by full-dose transmission data, i.e., ${T}_{\mathrm{uld}}={I}_0^{\mathrm{uld}}\times {T}_{\mathrm{sd}}$,
5. Simulating the noise in ultra-low-dose scan by adding Poisson noise and Gaussian noise to the transmission data, i.e., ${I}_{\mathrm{uld}}=\mathrm{Poisson}\left({T}_{\mathrm{uld}}\right)+\mathrm{Gaussian}\left({m}_e,{\sigma}_e^2\right)$,
6. Calculating ultra-low-dose projection data in the sinogram domain, i.e., ${p}_{\mathrm{uld}}=\log \left(\frac{I_0^{\mathrm{uld}}}{I_{\mathrm{uld}}}\right)$,
7. Reconstruction of the ultra-low-dose images using FBP algorithm,
8. Converting the reconstructed attenuation map to HU using the equation in step 1.

In the abovementioned steps for simulating ultra-low-dose scan, three parameters should be determined, namely, the ultra-low-dose scan incident flux (${I}_0^{\mathrm{uld}}$), the mean (*m*_*e*_), and the variance (${\sigma}_e^2$) of electronic noise. In modern CT scanners, these parameters can be determined during routine calibration procedures. However, this is not practical for multi-centric clinical database. Hence, these parameters were set based on the fitting noise level of the simulated ultra-low-dose CT images with a real ultra-low-dose CT image-set serving as a reference. The reference ultra-low-dose CT images were acquired under a task-specific ultra-low-dose protocol for the diagnosis of COVID-19 on the MX 16-slice CT scanner (Philips Healthcare) with a reduced CT dose index (CTDI_vol_) of about 0.72 mGy. The acquisition parameters of the protocol were as follows: tube potential of 90 kVp, tube current range of 20–45 mA, 0.5-s rotation time, and a pitch factor of 0.75 with the FBP image reconstruction procedure. To quantify the noise level of the simulated ultra-low-dose CT images, the noise index was produced based on the method proposed by Christianson et al [26]. The incident flux level (${I}_0^{\mathrm{uld}}$) was determined when the magnitudes of noise levels in soft-tissue and lungs between simulated ultra-low-dose images were within 10% interval compared with that in the reference images. In electronic systems, *m*_*e*_ is usually calibrated to be zero whereas the variance of electronic noise was initialized based on the method proposed by Zeng et al [27] for the Definition Edge CT scanner (Siemens Healthcare). Subsequently, an observer study was performed to evaluate the quality of simulated ultra-low-dose images against the full-dose images. Three physicists took part in this study to visually score the apparent Poisson noise and streak artifacts owing to statistical errors originating from low photon scanning and Gaussian noise. We categorized our dataset into multiple groups according to the scanner model and imaging protocol used. Consequently, three image sets were randomly selected from each group for the evaluation process. Two ROIs (5 × 5 cm^2^) were drawn in the soft-tissue (upper part of the liver) and lung regions without including adjacent anatomic structures. The average standard deviation (STD) across the ROIs was calculated. The simulation parameters were updated to obtain the same STD in two ROIs drawn on soft-tissue and lungs, while the visual similarity between simulated ultra-low-dose image and full-dose image was preserved.

### Deep learning algorithm

#### Network architecture

**Fig. 1:**
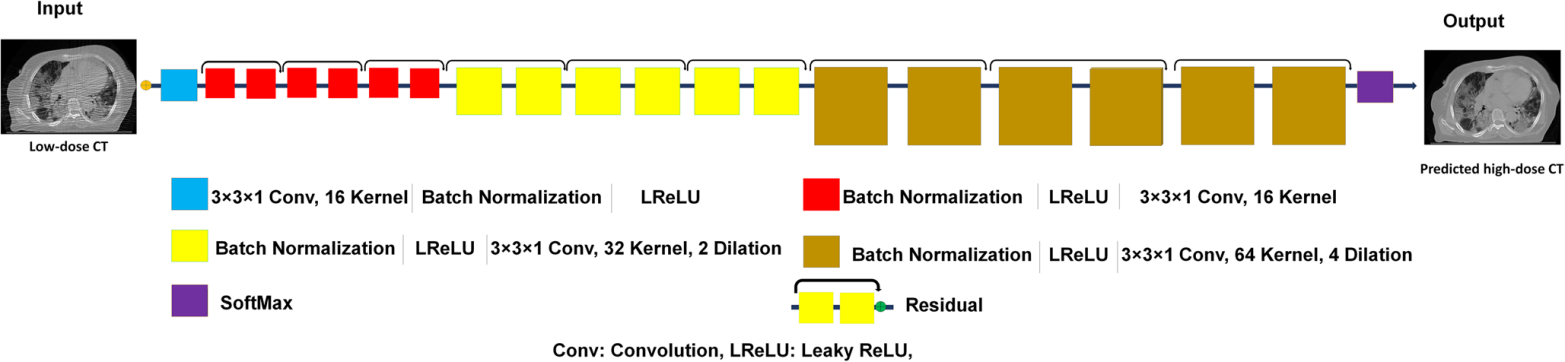
Architecture of the deep residual neural network (ResNet) along with details of the associated layers. Red color layer, layer with dilation 1; yellow color layer, layer with dilation 2; brown color layer, layer with dilation 4. Conv, convolutional kernel; LReLu, leaky rectified linear unit; SoftMax, Softmax function; Residual, residual connection

We applied a deep residual neural network (ResNet) for image to image transformation in an attempt to predict full-dose from ultra-low-dose CT images [28]. The residual model proposed by Wenqi et al [28] for image classification was modified for regression application in this study. Figure 1 presents the architecture of ResNet employed in the current study. This network combines 20 convolutional layers, including two seven and one six convolutional layers for low-, medium-, and high-level features extraction. For effective feature extraction, the ResNet architecture adopts a dilated convolution with factors of 2 and 4 for seven intermediate and six last layers. In this combination, every two convolutional layers are linked to a residual connection where a leaky rectified linear unit (LReLU) acts as an activation function. The ResNet implemented in TensorFlow (version 1.12.1) was utilized to transform ultra-low-dose to full-dose chest CT images.

In this work, a 3 × 3 × 1 kernel was applied for all convolutions. The ResNet network has residual connections that bypass the parameterized layers through combining the input and output of a block to render a smooth information propagation, thus enhancing the training speed/quality. The ResNet architecture benefits from 9 residual blocks that proved efficient for improving the feature extraction process. This work avoids a large number of trainable parameters. More detail of ResNet architecture is presented in Fig. 1.

#### Implementation details

In this study, 800 (112 COVID-19), 170 (100 COVID-19), and 171 (100 COVID-19) pairs of ultra-low-dose and full-dose CT studies were used as input/output as training, test, and external validation set, respectively, to implement the full-dose prediction technique. The ResNet model with an architecture of a 2D spatial window equal to 512 × 512voxels was employed (CT images were cropped to eliminate the bed and background air). To train the network, Adam optimizer and L2norm loss function were adopted. The training of the network for full-dose prediction took about 50 h using a 2080TI GPU, Intel(R) Xeon 2.30-GHz 7i CUP, and 64-GB RAM. After ten epochs, the training loss reached its plateau.

### Quantitative evaluation

Our qualitative and quantitative evaluation of the framework was performed on 170 tests and 171 external validation set. To this end, ultra-low-dose and predicted images were compared with reference full-dose images. The quality of CT images was assessed using voxel-wise root mean square error (RMSE). Moreover, the structural similarity index (SSIM) and peak signal-to-noise ratio (PSNR) were used as quantitative measures of the quality of the predicted CT images.

### Clinical evaluation

All patient chest CT images were categorized into three groups, namely full-dose, ultra-low-dose, and predicted by lung windowing. Blind qualitative assessment of CT images was performed by a radiologist with 10 years of experience. The radiologists’ clinical evaluations were based on qualitative assessment, including appraisal of lesion density, shape, position, and margin in addition to the analysis of lesion type. For the qualitative assessment, scores ranging from 1 to 5 were assigned to each image as follows: excellent, 5; good, 4; adequate, 3; poor, 2; and uninterpretable, 1. This scoring scheme was separately used for the overall assessment of image quality, i.e., margin, shape, and density as well as for lesion type. Lesion types included ground glass opacities (GGO), crazy paving (CP), consolidation (CS), nodular infiltrates (NI), bronchovascular thickening (BVT), and pleural effusion (PE). To categorize lesions based on their location, they were attributed to any of the following anatomical regions in the lung: left lung, right lung, upper zone, lower zone, middle zone, superior segment, posterior segment, and central and peripheral areas.

## Results

The mean value of CTDI_vol_ for the ultra-low-dose protocol based on which the simulation parameters are determined is about 0.72 mGy (range 0.66–1.02 mGy) (Table 1). In contrast, this index ranges from 4.16 to 10.5 mGy with an average of 6.5 mGy for the full-dose protocol. According to the adopted methodology, the incident flux was determined in the range of 3.5–4 × 10^3^ for different scanner models.

**Fig. 2:**
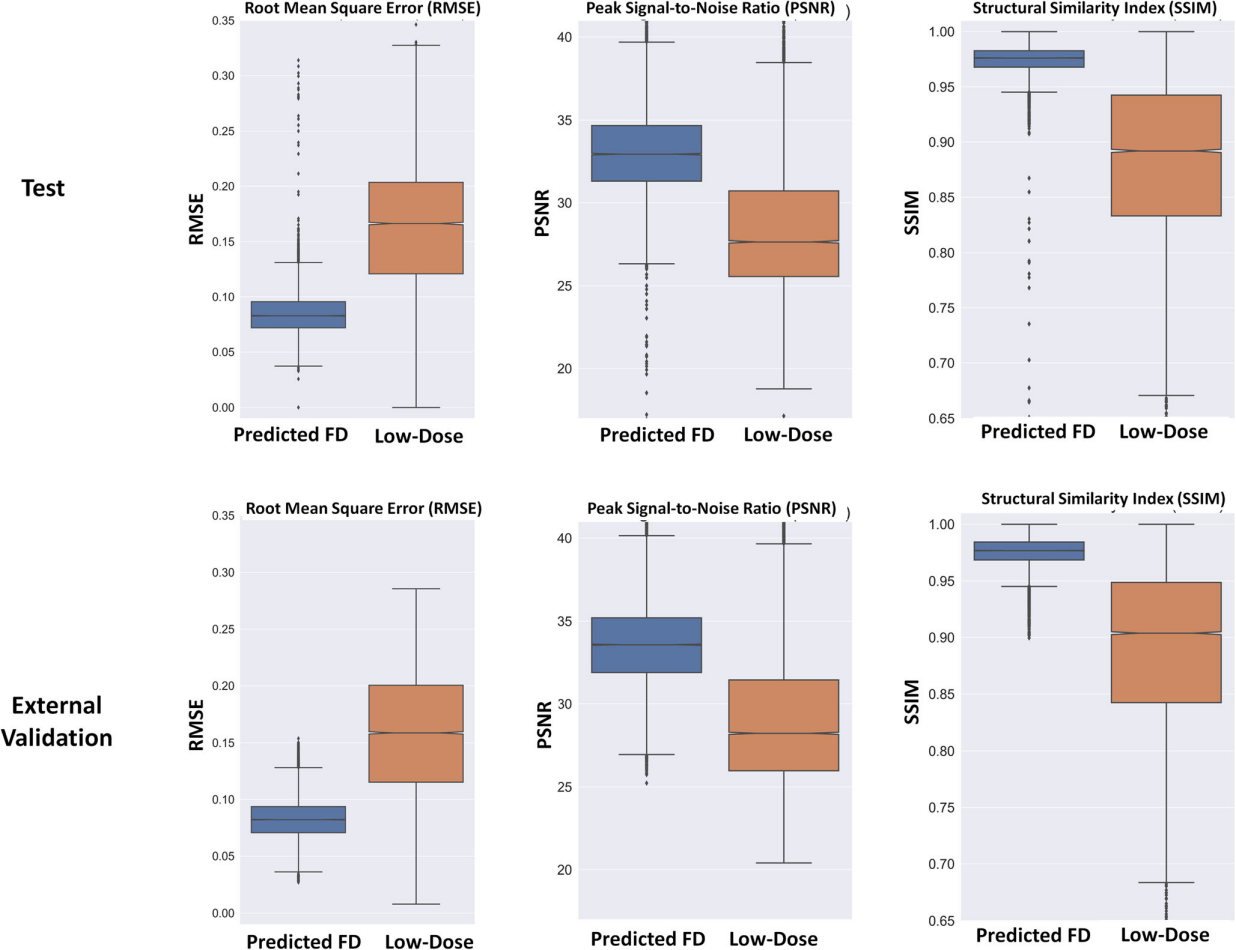
Mean and STD of peak signal-to-noise ratio (PSNR), structural similarity index (SSIM), and root mean square error (RMSE) for the predicted and ultra-low-dose CT images in the test and external validation sets

The quantitative metrics, including RMSE, PSNR, and SSIM for predicted full-dose and ultra-low-dose CT images in the test and external validation sets, are plotted as box plots in Fig. 2 and summarized in Table 2. The RMSE in units of normalized HU decreased from 0.16 ± 0.05 to 0.09 ± 0.02 and from 0.16 ± 0.06 to 0.08 ± 0.02 for predicted full-dose images from ultra-low-dose CT images in test and external validation set, respectively. The SIMM and PSNR increased from 0.89 ± 0.07 to 0.97 ± 0.01 and from 29.40 ± 4.94 to 33.60 ± 2.70 for predicted full-dose images in the external validation set, respectively.

**Table 2:** Mean and STD of peak signal-to-noise ratio (PSNR), structural similarity index (SSIM), and root mean square error (RMSE) for the predicted and ultra-low-dose CT images in the test and external validation sets and statistical difference between predicted and ultra-low-dose images

Parameters	Images	Test	External validation
RMSE	Predicted	0.09 ± 0.02	0.08 ± 0.02
	Ultra-low-dose	0.16 ± 0.05	0.16 ± 0.06
*p* value		*p* < 0.0001	*p* < 0.0001
PSNR	Predicted	32.97 ± 2.60	33.60 ± 2.70
	Ultra-low-dose	28.44 ± 3.87	29.40 ± 4.94
*p* value		*p* < 0.0001	*p* < 0.0001
SSIM	Predicted	0.97 ± 0.02	0.97 ± 0.01
	Ultra-low-dose	0.89 ± 0.07	0.89 ± 0.07
*p* value		*p* < 0.0001	*p* < 0.0001

**Fig. 3:**
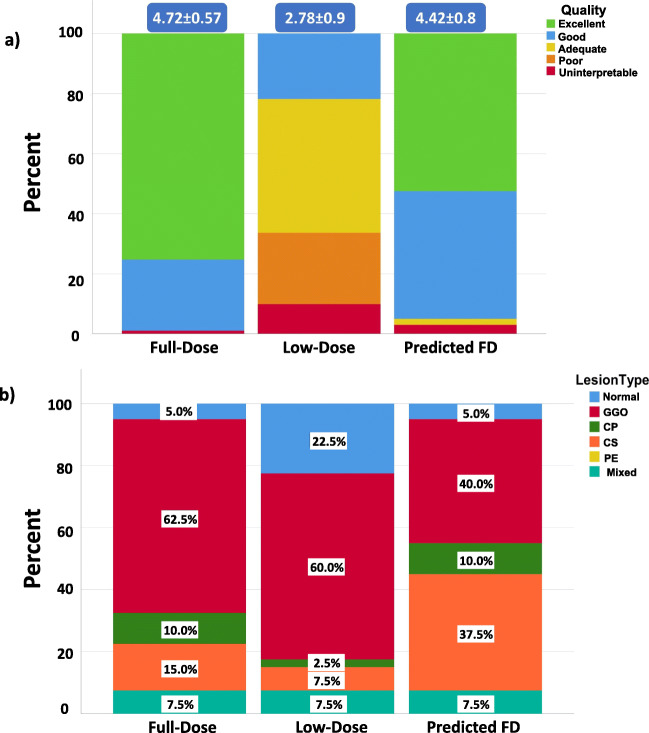
**a** Image quality scoring of different images. **b** Lesion type frequency in different images. Ground glass opacities (GGO), crazy paving (CP), consolidation (CS), nodular infiltrates (NI), bronchovascular thickening (BVT), and pleural effusion (PE). Scores (excellent, 5; good, 4; adequate, 3; poor, 2; and uninterpretable, 1)

The overall results associated with the assessment of image quality are shown in Fig. 3a wherein high image quality variations can be observed in ultra-low-dose scans, while the predicted full-dose images are mostly scored good or excellent. Overall scoring shows that the full-dose images received the highest score (4.72 ± 0.57) whereas the ultra-low-dose images were rated with the lowest scores (2.78 ± 0.9). In Fig. 3b, the frequency of occurrence of each lesion type in the different series of images is shown. As can be seen, GGO has the highest occurrence in all images, whereas mixed (all) had the same occurrence for all images. Changes in the essence of features are as follows: in the ultra-low-dose group, GGO is shifted to normal feature whereas consolidation is turned to GGO.

**Fig. 4:**
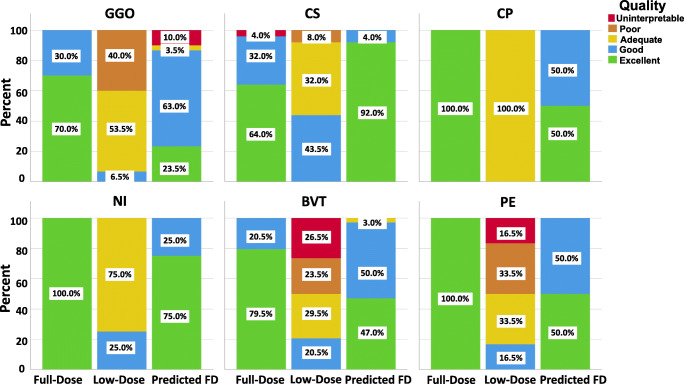
Image quality scoring of different images. Ground glass opacities (GGO), Crazy Paving (CP), Consolidation (CS), Nodular Infiltrates (NI), Bronchovascular thickening (BVT), and Pleural effusion (PE). Scores (excellent: 5, good: 4, adequate, 3, poor: 2 and uninterpretable: 1)

Lesion detectability scoring results are shown in Fig. 4. The excellent score (score = 5) for CS in full-dose images is in about 60% of the cases while it exceeds 90% in predicted full-dose CT images. CP, NI, and PE achieve an excellent score (100%) in predicted images and is more than 40%, 70%, and 40% of the cases, respectively. The overall image quality scores assigned by human observers for different lesions are summarized in Table 3. Table 4 presents the visual scoring of different images for different aspects of CT findings, including lesion status, margin, shape, and density.

**Table 3:** Image quality scores assigned by human observers for different lesions. *GGO*, ground glass opacities; *CS*, consolidation; *CP*, crazy paving; *NI*, nodular infiltrates, *BVT*, bronchovascular thickening; *PE*, pleural effusion (PE). Scores (excellent, 5; good, 4; adequate, 3; poor, 2; and uninterpretable, 1)

Lesions	Full-dose	Ultra-low-dose	Predicted
GGO	4.70 ± 0.47	2.67 ± 0.61	3.90 ± 1.09
CS	4.52 ± 0.87	3.36 ± 0.64	4.92 ± 0.28
CP	5.00 ± 0.00	3.00 ± 0.00	4.50 ± 0.71
NI	5.00 ± 0.00	3.25 ± 0.50	4.75 ± 0.50
BVT	4.79 ± 0.41	2.44 ± 1.11	4.44 ± 0.56
PE	5.00 ± 0.00	2.50 ± 1.05	4.50 ± 0.55

**Table 4:** Image quality assessment through visual scoring of different images documenting different aspects of CT findings. Scores (excellent, 5; good, 4; adequate, 3; poor, 2; and uninterpretable, 1)

CT findings			Full-dose	Low-dose	Predicted
Lesion status	Laterality	Left lung	4.66 ± 0.55	3.14 ± 0.69	4.52 ± 0.51
		Right lung	4.70 ± 0.53	3.12 ± 0.65	4.52 ± 0.51
	Cephalocaudal distribution	Upper	4.44 ± 0.63	2.94 ± 0.44	4.25 ± 0.45
		Lower	4.68 ± 0.54	3.10 ± 0.60	4.48 ± 0.51
		Middle	4.71 ± 0.53	3.23 ± 0.56	4.48 ± 0.51
	Location	Central	4.67 ± 0.58	3.33 ± 1.15	5.00 ± 0.00
		Peripheral	4.76 ± 0.44	3.12 ± 0.70	4.71 ± 0.47
		Superior	4.65 ± 0.59	3.25 ± 0.64	4.60 ± 0.50
		Posterior	4.68 ± 0.54	3.23 ± 0.62	4.65 ± 0.49
		Central and peripheral	4.63 ± 0.62	3.19 ± 0.54	4.44 ± 0.51
Margin		Ill defined	4.48 ± 0.75	2.30 ± 0.91	4.19 ± 0.56
		Well defined	4.67 ± 0.55	3.15 ± 0.60	4.93 ± 0.27
Shape	Nodular		5.00 ± 0.00	4.00 ± 0.00	5.00 ± 0.00
	Wedged		5.00 ± 0.00	3.33 ± 0.82	5.00 ± 0.00
	Elongated		4.00 ± 1.41	2.00 ± 1.41	4.50 ± 0.71
	Confluent		4.54 ± 0.66	3.00 ± 0.66	4.54 ± 0.51
Density	Part solid		4.83 ± 0.41	2.40 ± 1.14	3.60 ± 1.52
	Solid		4.60 ± 1.26	3.40 ± 0.70	4.80 ± 0.42
	Pure GGO		4.63 ± 0.49	2.79 ± 0.66	3.96 ± 1.27
	GGO and CS		5.00 ± 0.00	2.80 ± 0.84	4.40 ± 0.55

**Fig. 5:**
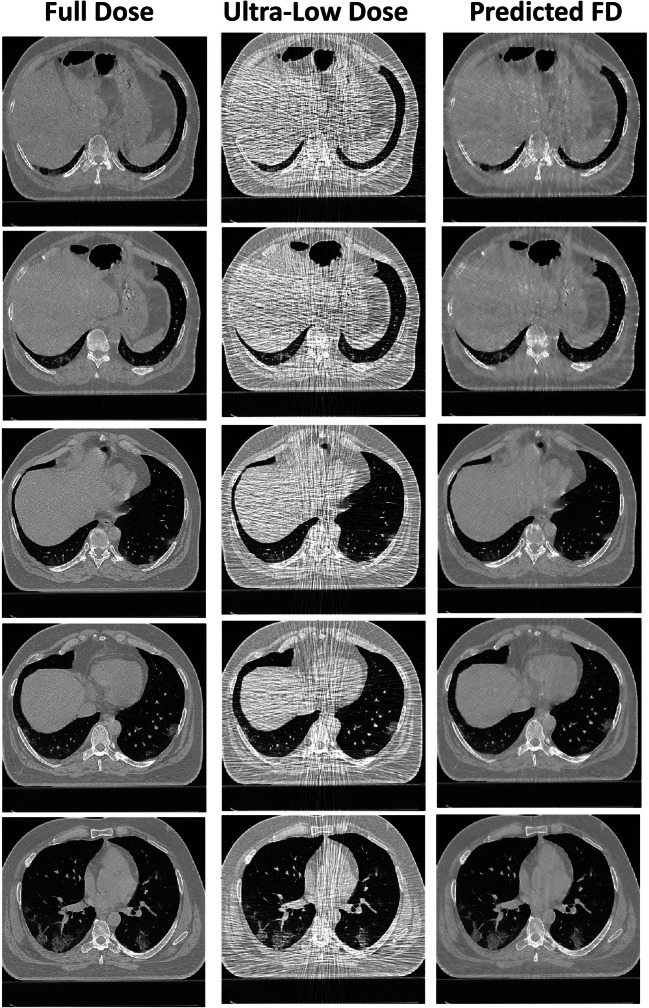
Representative full-dose image and corresponding ultra-low-dose and predicted full-dose images

**Fig. 6:**
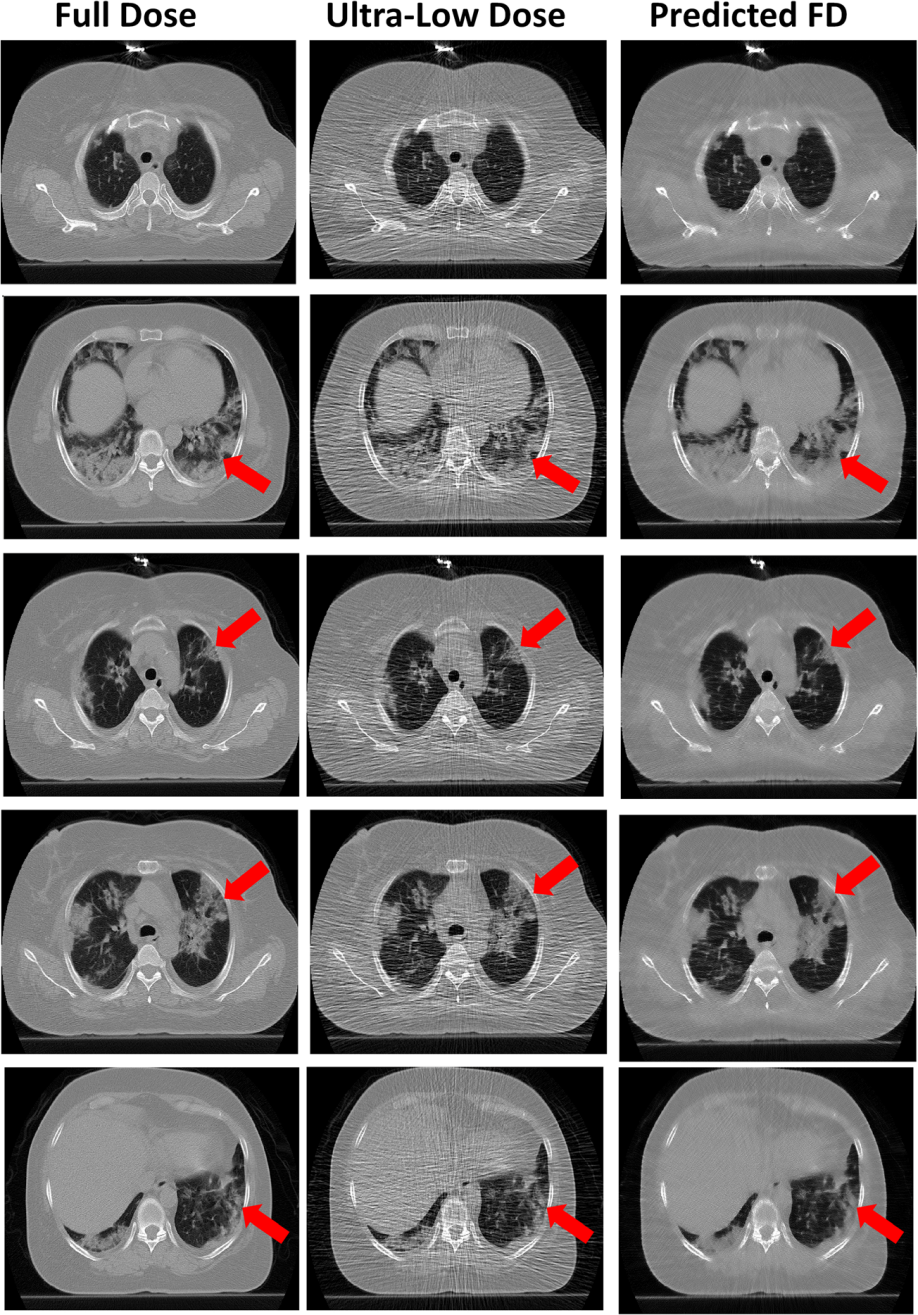
Outlier report: CT images of a patient where the deep learning algorithm improved image quality but changed the patchy lesion to consolidation in predicted images. The red arrows pinpoint changes in the identified lesions

Figure 5 and supplemental figures 1 and 2 present a representative example of a full-dose, ultra-low-dose, and predicted full-dose CT images. The predicted CT images improved image quality, thus enabling most lesions to be easily classified. Figure 6 and supplemental figures 3 and 4 show an example of an outlier in which image quality was improved; however, some relevant anatomical details were missing. Hence, the network failed to recover the full detail of images and GGO lesion converted to CS. For an outlier in the predicted group, GGO was shifted to consolidation.

## Discussion

Despite the controversies and heated debates around the potential haphazardous effects of low levels of ionizing radiation and the linear-no-threshold theory [29], concerns from radiation exposure are still current [30]. Since CT imaging is widely used in clinical diagnosis, prognosis, and assessment of response to treatment and follow-up of a number of diseases, it is an incremental source of radiation dose to patients in modern healthcare [7, 31]. With respect to the current COVID-19 crisis, chest CT imaging is the fastest diagnostic approach. However, it remains a high-dose imaging modality, and as such, developing an ultra-low-dose protocol enabling to maintain optimal image quality is clinically relevant in public health management. Therefore, as a response to this outbreak and the subsequent demand for CT imaging for mass population, an ultra-low-dose imaging approach was proposed to minimize radiation exposure of the population. This is achieved through a deep learning approach introduced for COVID-19 patient diagnosis by generating high-quality full-dose from ultra-low-dose CT images. It was shown that although the simulated ultra-low-dose CT images were diagnostically compromised, the generated full-dose images were appropriate for the task at hand. The proposed ultra-low-dose approach based on deep learning algorithms succeeded to reduce the CTDI_vol_ by up to 89%, reflecting a substantial reduction of the radiation dose associated with diagnostic CT examinations.

A number of studies have assessed the role of low-dose CT for COVID-19 management [11–14]. Agostini et al [12] evaluated the feasibility and diagnostic reliability of a low-dose, long-pitch dual-source chest CT protocol for COVID-19 patients in terms of signal-to-noise and contrast-to-noise ratio and Likert scales. They reported that their low-dose CT protocol achieved significant dose reduction, lower motion artifacts with optimum signal and contrast-to-noise ratio. However, this protocol is only applicable on third-generation dual-source CT scanners, and as such, it is not applicable on older CT imaging systems. Dangis et al [14] examined the accuracy and reproducibility of low-dose sub-millisievert chest CT for the diagnosis of COVID-19. They demonstrated that low-dose CT has excellent sensitivity, specificity, positive predictive value, negative predictive value, and accuracy for the diagnosis of COVID-19 with a mean effective dose of 0.56 ± 0.25 mSv. In the current study, the simulated ultra-low-dose CT images represent the outcome of a protocol with a significant reduction of CTDI_vol_ (up to 89%) compared with the corresponding full-dose CT images, which is a good metric for comparing patient effective dose and risks of ionizing radiation [32]. This is a commended effort in view of the current recommendations in radiation protection [33], particularly for the diagnosis and follow-up of a sensitive population, such as pediatric patients and pregnant women.

The results of this study demonstrated that by using CNNs, we could generate images with a significantly lower dose and acceptable image quality. Although image quality in the predicted images was not exactly identical to full-dose CT images, most COVID-19 features, including nodular infiltrate, consolidation, and crazy paving features, obtained high scores, almost similar to full-dose CT images.

We also demonstrated that the texture of COVID-19 lesions could be erroneously altered in the predicted CT images, which would skew the diagnosis/scoring. We observed that in the ultra-low-dose group, GGO was shifted to normal feature, whereas consolidation was shifted to GGO. In the low-dose group, the shift of GGO to normal features might be due to closeness of mean HU value of GGO to normal. In addition, as the differences between the HU value of GGO and consolidation lesions are located in the normal neighborhood, they may be depicted and diagnosed as similar features. Likewise, in the predicted group, GGO was shifted to consolidation owing to the local induced bias noise pattern in ultra-low-dose images, heterogeneity of lesions, and smoothing effect of deep learning in some outlier cases. The low-dose simulation would result in overall zero bias (zero-mean noise signal) with elevated noise variance depending on the underlying signals/textures and level of simulated low-dose scanning. Due to the fine texture as well as relatively low density (low CT numbers) of GGO lesions, the streak-like noise patterns led to mostly positive bias and rougher textures in these lesions. As such, the likelihood of misinterpretation of GGO with CP increased in the resulting synthetic standard dose CT images. In addition, the minimum widely used learnable kernel employed in the current study is 3 × 3, which would slightly smooth the structures of the resulting synthetic images. The local positive noise-induced bias along with the smoothness of the structures in the resulting CT images led to the misidentification of some GGO lesions with CP*.*

Although ultra-low-dose CT can be equally effective in COVID-19 detection and diagnosis as the full-dose CT, it suffers from a number of limitations, particularly the increased noise level caused by photon deprivation. One of the limitations of the present study was that during the clinical assessment, the ultra-low-dose images could be easily identified by radiologists because of the high noise present. This might have led them to be subconsciously biased, hence assigning lower scores to these images. We reported outliers originating mostly from the low quality of the simulated ultra-low-dose CT images (high noise level and/or noise-induced artifact) caused by photon starvation in simulated corpulent patients. The application of the current method in COVID-19 imaging warranted a thorough investigation of outliers owing to inter-/intra-patient variation and noise variability.

## Conclusion

Ultra-low-dose CT imaging of COVID-19 patients would result in the loss of critical information about lesion types. However, the results presented in this work indicated that ResNet is an optimal algorithm for generating ultra-low-dose CT images for COVID-19 diagnosis. Nevertheless, the deep learning solution failed to recover the correct lesion structure/density for a number of patients and as such, further research and development is warranted to address these limitations.

## Electronic supplementary material

ESM 1 (DOCX 3701 kb)

## Abbreviations

ADMIRE: Advanced modeled iterative reconstruction
AEC: Automatic exposure control
BVT: Bronchovascular thickening
CNN: Convolutional neural network
CNR: Contrast-to-noise ratio
COVID-19: Coronavirus disease 2019
CP: Crazy paving
CS: Consolidation
CT: Computed tomography
CTDI: CT dose index
DLP: Dose-length product
ED: Effective dose
FBP: Filtered backprojection
GAN: Generative adversarial network
GGO: Ground glass opacities
NI: Nodular infiltrates
PE: Pleural effusion
RT-PCR: Real-time reverse transcription-polymerase chain reaction
SARS: Severe acute respiratory syndrome
SARS-CoV-2: Severe acute respiratory syndrome coronavirus 2
SNR: Signal-to-noise ratio
WHO: World Health Organization
