# Supplementary material for: Ultra-low-dose chest CT imaging of COVID-19 patients using a deep residual neural network
Source: Eur Radiol. 2020 Sep 3;31(3):1420–31. doi: 10.1007/s00330-020-07225-6 (PMC7467843; doi:10.1007/s00330-020-07225-6)
Supplement: Supplementary file 1 — (DOCX 3701 kb) [file 330_2020_7225_MOESM1_ESM.docx]

**
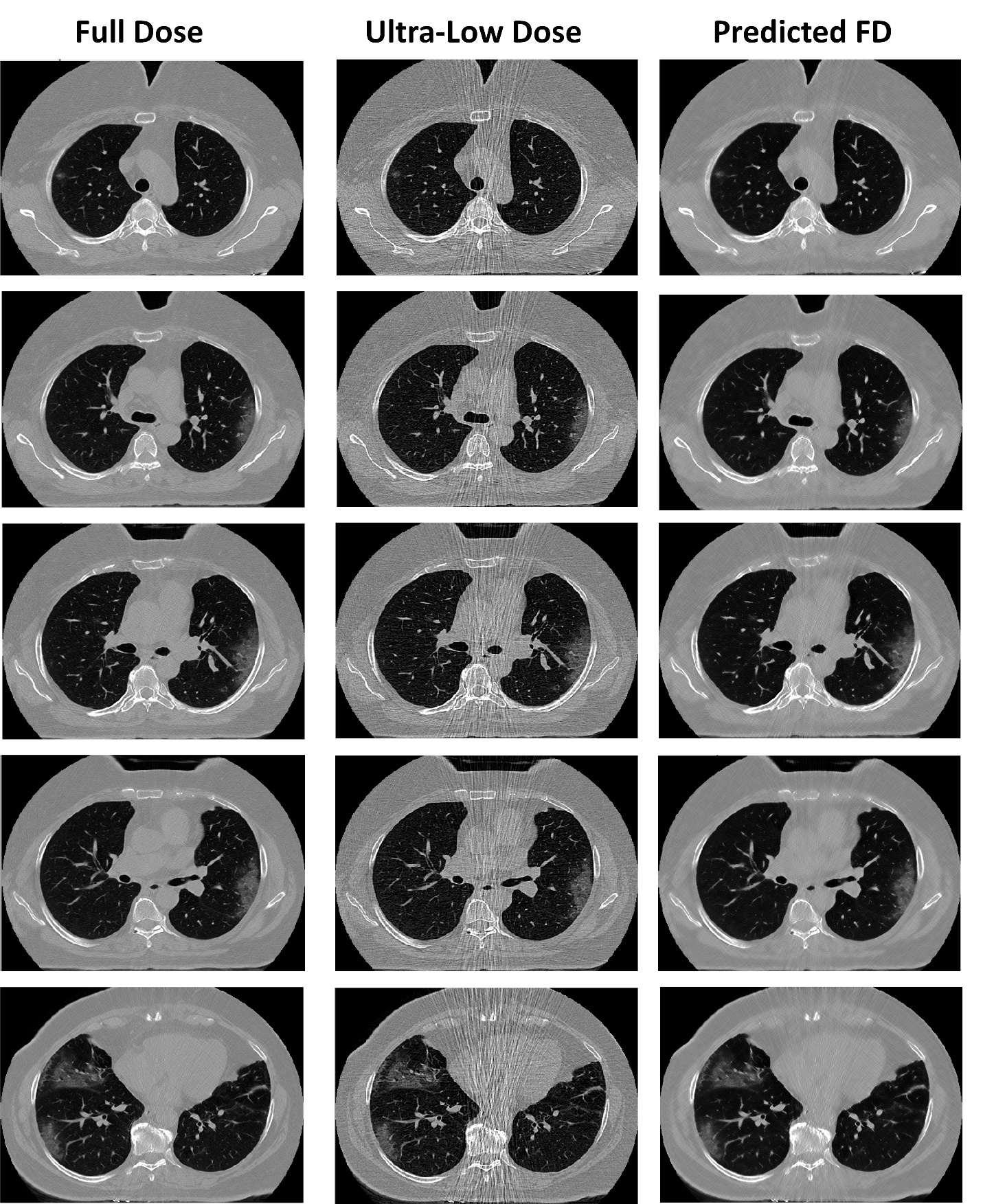
**

**Supplemental Figure 1.** Representative full-dose image and corresponding ultra-low-dose and predicted full-dose images case 2.

**
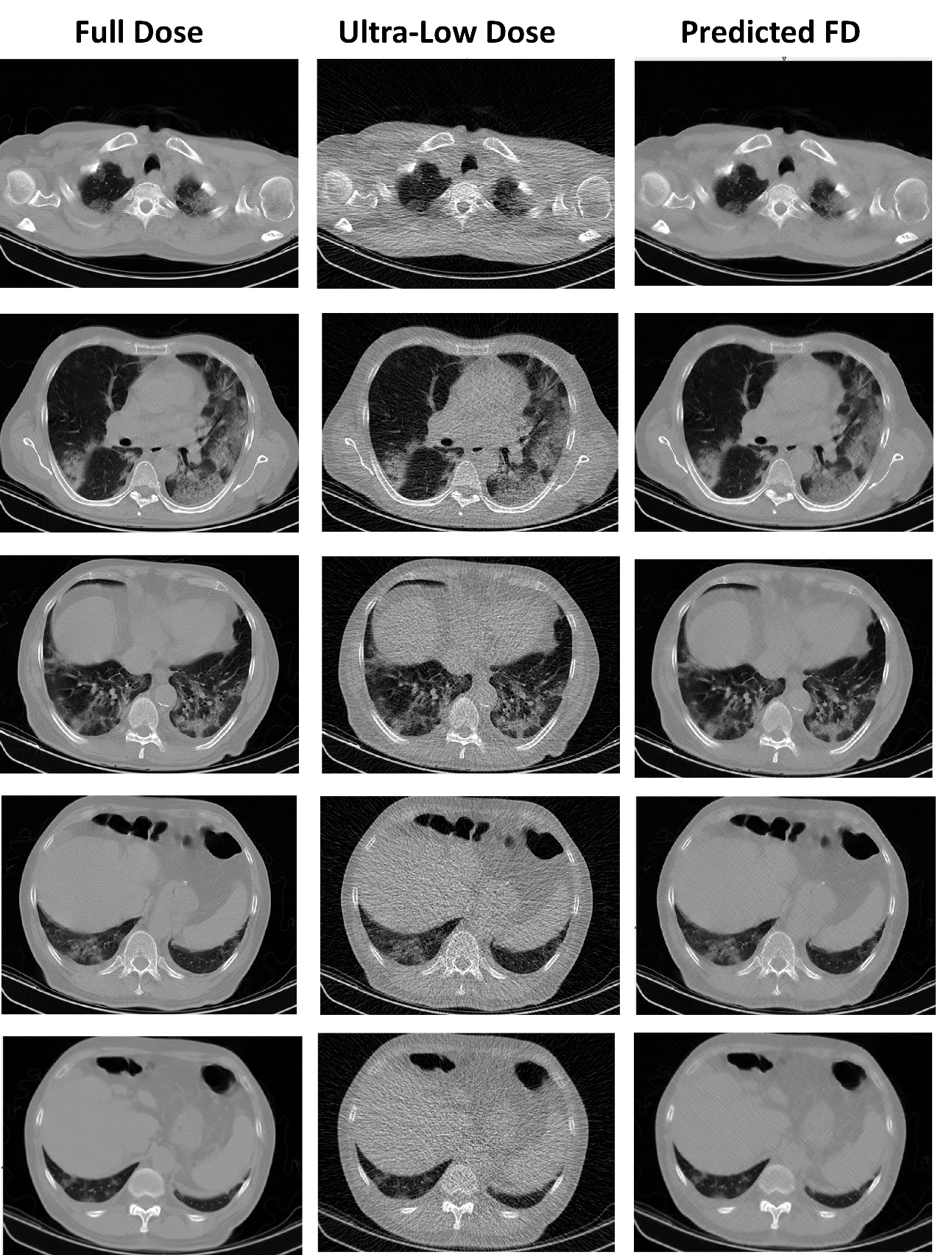
**

**Supplemental Figure 2.** Representative full-dose image and corresponding ultra-low-dose and predicted full-dose images case 3.


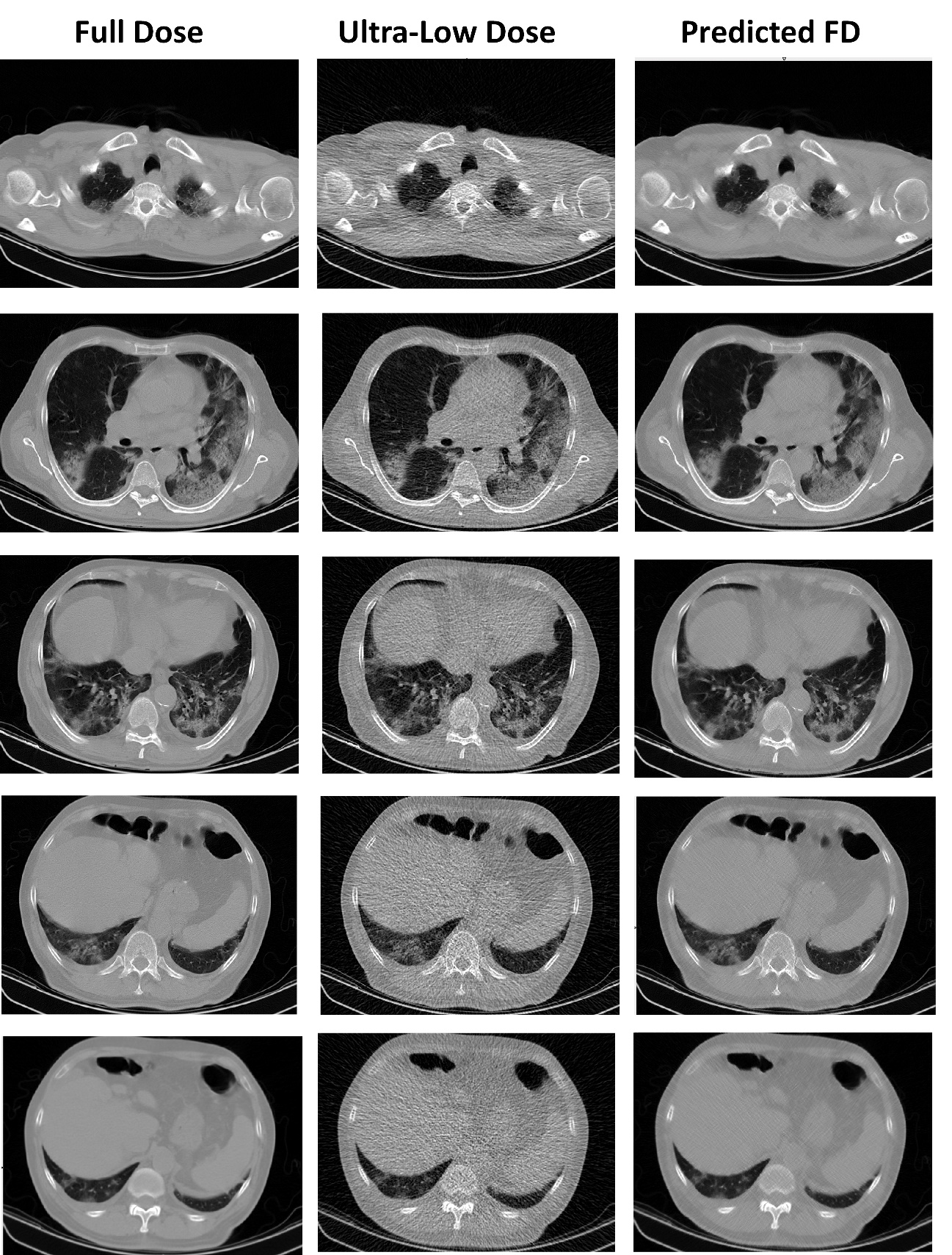


**Supplemental** **Figure 3.** Outlier report: CT images of a patient where the deep learning algorithm improved image quality but changed the patchy lesion to consolidation in predicted images case 2.

**
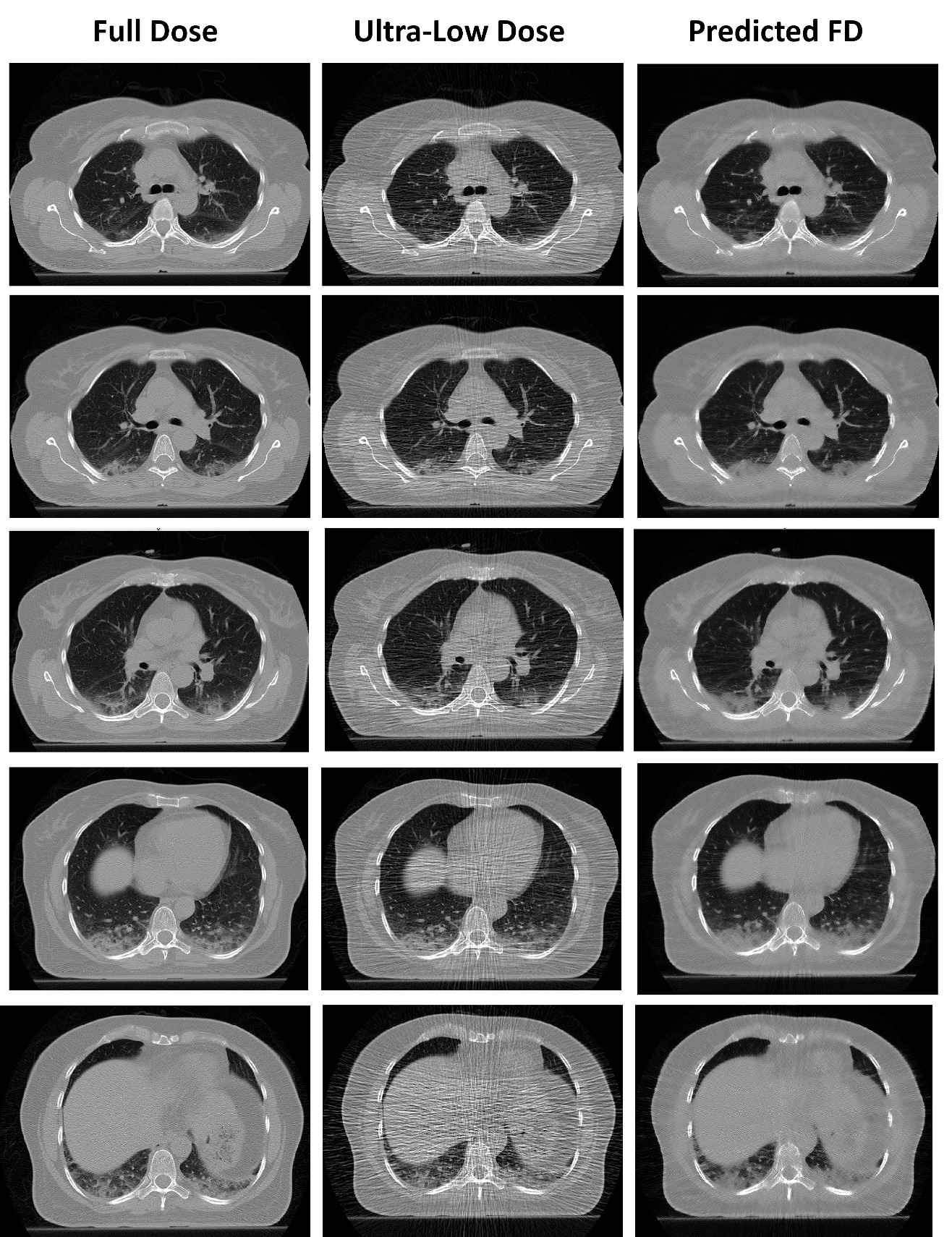
**

**Supplemental** **Figure 4.** Outlier report: CT images of a patient where the deep learning algorithm improved image quality but changed the patchy lesion to consolidation in predicted images case 3.
